# Effectiveness of the Cyriax Technique With Conventional Therapy in the Management of Lateral Epicondylitis: A Case Report

**DOI:** 10.7759/cureus.61813

**Published:** 2024-06-06

**Authors:** Aditi Nagore, Snehal Samal, Pradhyum D Kolhe

**Affiliations:** 1 Musculoskeletal Physiotherapy, Ravi Nair Physiotherapy College, Datta Meghe Institute of Higher Education and Research, Wardha, IND; 2 Neurophysiotherapy, Ravi Nair Physiotherapy College, Datta Meghe Institute of Higher Education and Research, Wardha, IND

**Keywords:** exercises, deep transverse friction massage, conventional therapy, cyriax technique, lateral epicondylitis, extensor carpi radialis brevis

## Abstract

Tennis elbow, medically referred to as lateral epicondylitis, is a common musculoskeletal condition that results in tenderness and pain on the outer side of the elbow. Physiotherapy is a conservative therapy for treating tennis elbow that emphasizes pain control, functional improvement, and recurrence avoidance. The case report examines the case of a 25-year-old woman who experienced a gradual onset of pain and mild swelling in her right elbow. As a result, she had hand trembling during flexion and extension, which was ongoing for the previous two days. As per the case study results, physical therapy has a crucial role in enhancing the endurance of muscles, increasing the range of motion, facilitating regular activities, and improving overall quality of life. According to recently published articles, a physical therapist plays a critical role in healing overuse injuries and helping patients resume their regular activities. For this patient, we developed a four-week treatment plan that includes several advanced therapy approaches, such as the Cyriax technique. The specific intervention used in the treatment was the Cyriax technique, which included intense deep friction massage followed by Mill's manipulation in addition to conventional physiotherapy which includes eight minutes of clinical ultrasound, isometric wrist joint stretches, praying position stretches, stretching exercises, and ice fomentation). For a total of 12 sessions, four weeks, the patient received treatment three days a week. For our patient, who had right-hand lateral epicondylitis, we designed a comprehensive rehabilitation program, and it was remarkably effective. We assessed the efficacy of our end measures using a variety of outcomes, including the Visual Analogue Scale (VAS) and the Patient-Rated Tennis Elbow Evaluation (PRTEE) scale. It was shown that giving patients a Cyriax method approach in addition to a regular physiotherapy treatment would be more advantageous for enhancing their general health and quality of life. To treat lateral epicondylitis, physiotherapists use a variety of manual treatments, such as mobilization, manipulation, and massage.

## Introduction

The extensor tendons in the dorsal forearm that is attached to the lateral epicondyle of the humerus are affected by the debilitating condition known as lateral epicondylitis. Tennis elbow, lateral epicondylitis, tendonitis, or other similar conditions are conditions that mostly affect the radiohumeral joint which causes continuous pain over the elbow. It is commonly observed across various professions and hobbies, although it is frequently linked to repetitive movements, like those found in racquet sports. Additional rapid, difficult, continuous eccentric movements and gliding joint grasping motions tend to be contributing factors. Usually, the dominant arm is impacted. The incidence of lateral epicondylitis is 3.4 per 1,000 patients. In patients who are 40-49 years old and 50-59 years old, respectively, the incidence is higher [1]. The average number of episodes of epicondylitis occurs during the span of two years and six months. Lesions within the extensor carpi radialis brevis (ECRB) muscle manifest as both macroscopic and microscopic abnormalities in cases of tennis elbow. The Patient-Rated Tennis Elbow Evaluation (PRTEE) scale and the Visual Analogue Scale (VAS) were utilized to assess the efficacy of the treatment on pain and functional enhancement, respectively. The damaged tendon in around 90% of instances is the ECRB muscle [2]. Discomfort in the area surrounding the outer elbow may be diagnosed as periostitis, lateral epicondylitis, epicondylalgia, tennis elbow, or ECRB tendinosis [3]. It's a prevalent root of elbow pain, with a yearly prevalence of between 10 and 30 occurrences for every 1,000 peaking between the ages of 35 and 55 [4]. Lesions of both microscopic and macroscopic nature can be observed in the ECRB in individuals suffering from tennis elbow. Chronic discomfort and tenderness in the outer elbow area are the primary indicators of tennis elbow, causing even basic actions such as picking up a cup of coffee or shaking hands to be quite painful [5]. Tennis elbow is not just painful; it can also have long-term consequences that make it difficult for a person to go about their everyday life and employment [6]. Primary complaints and major clinical presentation of tennis elbow include increased pain, decreased ability to grasp, and decreased physical activity. These symptoms can significantly affect everyday activities [7]. It's simple to diagnose tennis elbow, and a test that causes discomfort and tenderness when the lateral end of the epicondyle aspect is palpated, as well as resistance to wrist extension, resistance to middle finger extension, and passive wrist flexion, can confirm the diagnosis [8]. To assess specific conditions, Mill's test involves gently rotating the patient's forearm, completely bending the wrist, straightening the elbow, and subsequently examining the lateral epicondyle through palpation. In the second Cozen test, the examiner secures the patient's elbow by applying pressure to the lateral epicondyle with the thumb, ensuring stability.

The primary factor contributing to this disease is a degenerative mechanism triggered by excessive use of the common extensor tendon and the ECRB [9,10]. Based on different histopathological investigations that documented the condition's microscopic look and characteristics, the word had previously been defined as angiofibroblastic dysplasia. During ultrasound scanning, it is common to come across calculations, tears within the substance, notable irregularities in the lateral epicondyle, as well as variations in the thickness and composition of the common extensor tendon [11]. Cyriax has reported notable advancements in the treatment of tennis elbow through the application of deep transverse friction (DTF) in combination with Mill's manipulation, which is performed immediately following DTF [12]. To be recognized as a Cyriax intervention, it is essential to utilize the two components in the specified sequence. Participants are required to complete therapy sessions three times per week for a duration of four weeks [13]. DTF is a specialized form of therapeutic massage that focuses on treating soft tissue structures such as tendons [14]. Friction massage is employed to hinder the development of adherent scars and preserve the flexibility of the soft tissue structures encompassing muscles, ligaments, and tendons. Deep tissue massage differs from superficial massage in terms of technique and target tissue, while superficial massage is performed along the length of the blood vessels to enhance circulation and fluid movement. The objective of the superficial massage is to apply light pressure when beginning your massage. This approach aims to improve circulation and warm up the tissues in the treatment area, readying the body for the upcoming deeper procedures. Deep tissue massage objectives entail targeting the inner layers of your muscles and connective tissues with slow, deep strokes to deliver continuous pressure. This helps in releasing tension from muscles and tissue and breaks up scar tissue that develops after an injury [15].

## Case presentation

On the 6th of March 2024, a female badminton player, aged 25, visited the physiotherapy outpatient department at a private hospital. She presented with a chief complaint of experiencing pain and swelling on the lateral side of her right elbow, which is the extensor side. She also reported that she had been experiencing trembling in her right wrist and forearm while performing flexion and extension activities for the past five days. Based on the patient's account, it was reported that on the 5th of March 2024, she displayed tremors for 5-10 mins in her wrist while attempting to lift heavy items. Additionally, she encountered pain on the outer side of her elbow, which initially developed gradually and extended towards the hypothenar eminence of her palm. Pain is typically worse when working, at night, and during periods of relaxation before becoming better. She underwent some specific elbow tests (Mill and Cozen) as part of the investigation, and tennis elbow was determined to be her diagnosis. Following the patient's written consent, the treatment interventions were initiated on the first day that is from the 7th of March 2024. Mill's manipulation and intense friction massage were the specific interventions used for the treatment, along with eight minutes of clinical ultrasound in Cyriax physiotherapy. Twelve sessions, or three days a week, of treatment were given to the patient over the course of four weeks.

Clinical findings

A physical examination revealed swelling in the right elbow's extensor origin and the top third of the forearm. The level of discomfort was assessed as tolerable at a 7 on a VAS. Palpation revealed tenderness on the lateral epicondyle of the humerus, causing the patient to wince and withdraw in pain. This indicates a grade 3 level of tenderness in the affected area. The palpable trigger point, a highly distressing knot within the tense bands of the skeletal muscle fascia, could be felt at the same extensor origin. On the first day of physiotherapy, written consent from the patient was obtained before any procedures started. The treatment involved a particular intervention that combined the Cyriax technique with eight minutes of clinical ultrasound. The treatment plan consisted of 12 sessions spread out over a period of four weeks, with the patient attending therapy thrice a week, every other day [16]. There was muscle weakness in the right upper limb compared to the left. The right elbow and wrist on the affected side exhibited significant limitations in their range of motion (ROM), leading to discomfort during any type of movement. The timeline of the above event is shown in Table 1. 

**Table 1:** Timeline of the events AVBRH: Acharya Vinoba Bhave Rural Hospital; OPD: outpatient department

Evaluation	Date
Visited AVBRH at physiotherapy OPD	6th of March 2024
Symptom arousal	5th of March 2024
Starting of the physiotherapy sessions	7th of March 2024

Manual muscle testing (MMT) scales were utilized to assess the patient's condition. The findings from the MMT conducted prior to and following the treatment are presented in Table 2.

**Table 2:** MMT: pre- and post-treatment (the evaluation of the strength of affected muscles using the modified Oxford grading system) MMT: manual muscle testing; 3+ (fair+): holds test position against slight pressure; 4+ (good+): holds test position against moderate to strong pressure

Upper limb joints	Right (pre-treatment)	Right (post-treatment)
Elbow flexors	3+	4+
Extensors	3+	4+
Pronators	3+	4+
Supinators	3+	4+
Wrist flexors	3+	4+
Extensors	3+	4+
Radial deviation	3+	4+
Ulnar deviation	3+	4+

Further, Table 3 illustrates the ROM outcomes prior to and following the treatment. 

**Table 3:** ROM: pre- and post-treatment ROM of the affected (right) elbow and wrist ROM: range of motion

Joints	Right (pre-treatment)	Right (post-treatment)
Elbow flexors	0-120°	0-130°
Elbow extensors	0°	0°
Elbow pronators	0-70°	0-80°
Elbow supinators	0-60°	0-70°
Wrist flexors	0-65°	0-75°
Wrist extensors	0-63°	0-72°
Radial deviation	0-15°	0-25°
Ulnar deviation	0-22°	0-35°

Table 4 illustrates a comprehensive physiotherapy protocol that encompasses a full range of management strategies.

**Table 4:** Physiotherapy management protocol MHz: megahertz; W/m^2^: weber/square metre; sec: seconds; reps: repetition

Intervention	Dosage	Rational
Conventional treatment ultrasound: slow circular motion, continuous mode, frequency of 3 MHz, and intensity level of 1 W/m^2^	Over four weeks, participants will engage in a total of 12 sessions, with each session occurring three times per week. These sessions will take place on alternate days and will last for a duration of eight minutes each	It helps in reducing pain by increasing tissue temperature and promoting blood flow. By raising cellular activity and collagen synthesis, two essential processes for mending injured tendons, the intensity level facilitates tissue recovery. Because the ultrasonic waves are distributed evenly by the slow circular motion, the muscle and tendon fibres surrounding the elbow become more flexible and elastic
Isometric stretches for wrist joint	(3-5 sec hold for 10 reps once daily session)	It helps reduce pain by activating muscles without altering their length, reducing strain on damaged tendons, improving blood flow to the tendons and muscles, encouraging recovery, and lowering inflammation
Stretching exercises	(10-30 sec hold for 10 reps × daily session)	It helps by increasing blood flow and decreasing inflammation to assist and relieve pain, increase muscle endurance, and encourage tendon healing
Ice fomentation	(30 sec hold for 10 stretches × daily session)	The application of cold benefits in reducing discomfort, edema, metabolic rate, and blood vessel constriction
Cyriax technique	12 sessions × 3 times × 4 weeks which last for 10 minutes each will be conducted	Deep transverse friction massage is used in the Cyriax treatment for lateral epicondylitis in order to break down scar tissue, enhance blood flow, and encourage tissue repair. This approach targets the underlying pathophysiology in the injured tendons with the goals of relieving pain, decreasing inflammation, and restoring function

Physiotherapy management

**Figure 1:**
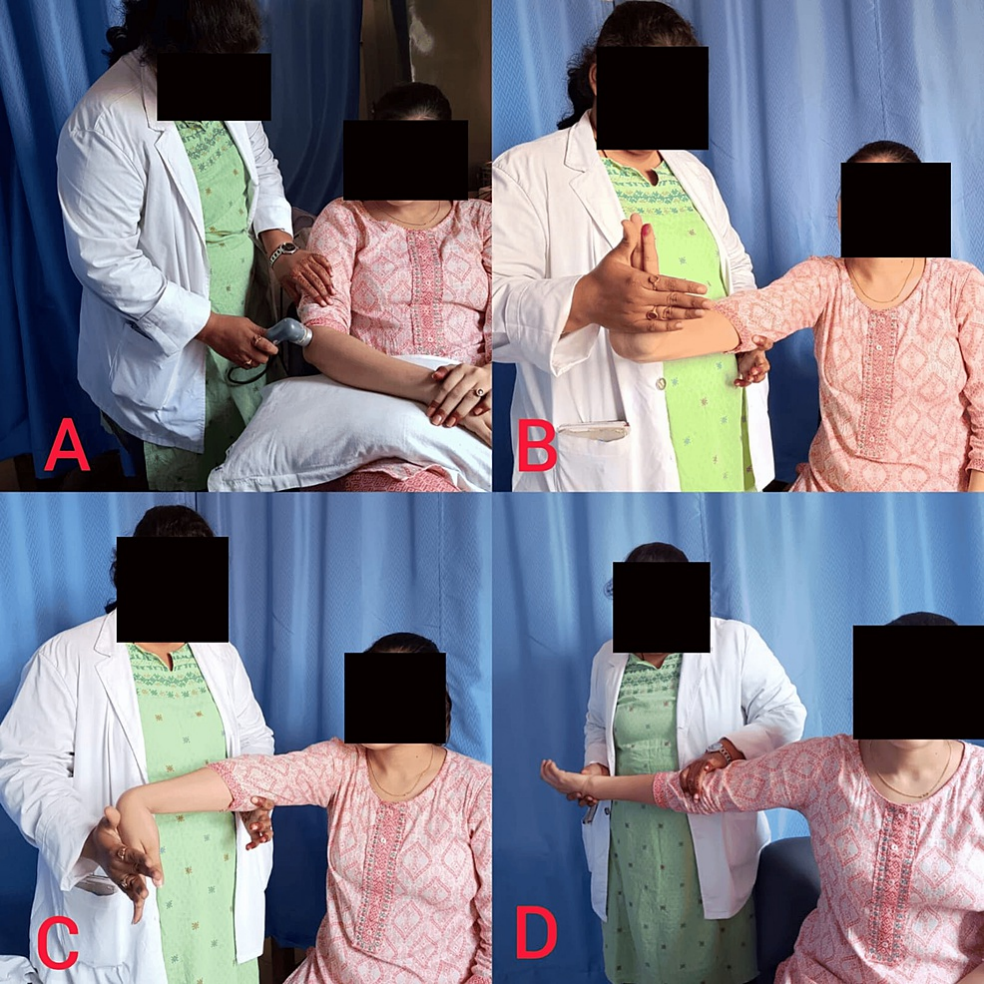
(A) Ultrasound. (B) Wrist extension stretching. (C) Wrist flexion stretching. (D) Cyriax technique

The standard treatment plan for managing lateral epicondylitis involves a variety of therapeutic methods designed to reduce pain and improve function. Ultrasound therapy utilizes slow circular motions at a frequency of 3 MHz and an intensity of 1 W/m^2^ (Figure 1A) [17]. Participants will have 12 sessions spread over four weeks, with each session occurring three times a week on alternate days, lasting eight minutes each. Isometric stretches focusing on the wrist joint, held for 3-5 seconds for 10 repetitions once a day, help enhance flexibility and strength (Figure 1B, 1C). In addition, stretches like the praying position and general stretching exercises, held for 10-30 seconds for 10 repetitions per daily session, aid in improving joint mobility and reducing stiffness. Ice therapy, held for 30 seconds for 10 stretches per daily session, helps reduce inflammation and provide temporary pain relief. The Cyriax technique, involving 12 sessions three times a week over four weeks, each lasting 10 minutes, complements the treatment plan by addressing specific musculoskeletal issues with specialized manual therapy techniques (Figure 1D). Together, these methods create a comprehensive approach to managing pain and dysfunction. 

Outcome measure

The outcomes evaluated by VAS after management follow-up are presented in Table 5.

**Table 5:** Follow-up and outcome VAS: Visual Analogue Scale; PRTEE: Patient-Rated Tennis Elbow Evaluation

Outcome measure	Pre-treatment	Post-treatment
VAS	7/10	3/10
PRTEE scale	55/100	30/100

## Discussion

Physiotherapy holds a crucial position in the holistic approach to treating tennis elbow, a prevalent overuse injury that specifically targets the tendons in the elbow region. Various techniques have been applied to address the symptoms and promote recovery. One of the most popular methods for treating injuries is eccentric training, which is gradually stretching the damaged muscle to improve its strength and flexibility. Reduced discomfort and improved function have been observed using this method, which promotes tissue remodeling and speeds up the healing process.

Manual therapy methods including soft tissue mobilization and joint mobilization are also beneficial in relieving muscular tension, increasing joint mobility, and decreasing discomfort. These techniques are commonly utilized in comprehensive physical therapy regimens that prioritize an overall strategy and are modified to meet the specific demands of each patient [18]. The objective of this study aimed to evaluate and contrast the impact of the Cyriax technique and conventional therapy on the grip strength, functional ability, and discomfort experienced by individuals diagnosed with lateral epicondylitis. The discomfort experienced by patients within the past 24 hours is assessed through a 10-point VAS, where individuals rate their level of discomfort on a scale ranging from 0 to 10. Research has demonstrated that it serves as a reliable indicator of both present pain levels and the likelihood of future pain intensity.

The evaluation of the participant's function and level of discomfort from the previous week was conducted using the PRTEE scale. Prior to and following the four-week course of therapy, all outcome measurements were taken. Over the span of four weeks, the therapy program involved a total of 12 sessions, with each session taking place three times a week [19]. The patient's experience serves as a prime illustration of the renowned healing abilities possessed by physiotherapists. In this particular case, the patient underwent manipulations and received treatment through the application of a therapeutic ultrasound electrical mode, showcasing the effectiveness of these techniques. Thus, we concluded that elbow conditions such as lateral epicondylitis can be effectively treated using manipulations [20].

## Conclusions

Tennis elbow can cause pain and decreased grip strength, which might interfere with the patient's activity of daily living. A physiotherapist plays an important role in treating overuse injuries of this kind and helping patients return to their regular activities. Participants with lateral epicondylitis showed notable decreases in pain, functional limitation, and grip strength when the Cyriax approach was used with regular physiotherapy following four weeks of daily therapy. This finding shows the use of the Cyriax technique in specific therapeutic or performance-oriented situations, in addition to its obvious advantages for anyone aiming to strengthen their grasp.
